# Research on the Resource Recovery of Medium-Chain Fatty Acids from Municipal Sludge: Current State and Future Prospects

**DOI:** 10.3390/microorganisms12040680

**Published:** 2024-03-28

**Authors:** Yuhao Liu, Yacong Duan, Long Chen, Ziyan Yang, Xiaoli Yang, Shuli Liu, Gangfu Song

**Affiliations:** School of Environmental and Municipal Engineering, North China University of Water Resources and Electric Power, Zhengzhou 450046, China; duan1218716691@163.com (Y.D.); 201613521@stu.ncwu.edu.cn (L.C.); yangziyan@ncwu.edu.cn (Z.Y.); yangxiaoli@ncwu.edu.cn (X.Y.); liushuli@ncwu.edu.cn (S.L.); sgf@ncwu.edu.cn (G.S.)

**Keywords:** municipal sludge, anaerobic fermentation, medium-chain fatty acids, hydrogen production, methane production

## Abstract

The production of municipal sludge is steadily increasing in line with the production of sewage. A wealth of organic contaminants, including nutrients and energy, are present in municipal sludge. Anaerobic fermentation can be used to extract useful resources from sludge, producing hydrogen, methane, short-chain fatty acids, and, via further chain elongation, medium-chain fatty acids. By comparing the economic and use values of these retrieved resources, it is concluded that a high-value resource transformation of municipal sludge can be achieved via the production of medium-chain fatty acids using anaerobic fermentation, which is a hotspot for future research. In this study, the selection of the pretreatment method, the method of producing medium-chain fatty acids, the influence of the electron donor, and the technique used to enhance product synthesis in the anaerobic fermentation process are introduced in detail. The study outlines potential future research directions for medium-chain fatty acid production using municipal sludge. These acids could serve as a starting point for investigating other uses for municipal sludge.

## 1. Introduction

Fossil fuels have been the primary source of energy since the beginning of the industrial revolution. Not only are fossil fuels a non-renewable resource but, more importantly, the continued growth of the global economy and population is leading to a shortage of fossil fuels. In addition, their increased burning is causing serious environmental problems, such as increased greenhouse gas emissions and global warming. There is an urgent need to find sustainable ways to meet global energy demand, mitigate climate change, and reduce pressure on air quality [1,2,3]. Thus, it is crucial to create technologies that produce energy while limiting the damaging effects that the production of organic waste has on the ecosystem.

With an expected increase to 97.72 million tonnes in 2023 [4], China’s sludge production is on the rise. Sludge is rich in nitrogen, phosphorus, and other trace elements, and contains a large amount of water. Municipal sludge is problematic because of its high water content; its abundance of nitrogen, phosphate, and other trace elements; its strong odour; the presence of hazardous compounds and pathogenic microbes; and its increased concentration of tiny particles. Secondary contamination of soil and groundwater can result from improperly managed municipal sludge, presenting a major threat to human and environmental health. The treatment and disposal of municipal sludge has long been a challenge and a focus of research in the environmental field, especially with the expansion of municipalities’ sewage treatment capacities and the rapid increase in sludge output [5].

The rational use of sludge as a resource is now one of the most pressing issues facing modern society. The resource utilisation of sludge, particularly through anaerobic fermentation, is of great importance in the context of fossil fuel energy scarcity. Anaerobic fermentation can improve sludge dewatering performance, reduce the number of pathogenic bacteria, and produce renewable energy, such as hydrogen (H_2_), methane (CH_4_), fatty acids (FAs), etc., to achieve sludge reduction and stabilisation, render the sludge harmless and make use of the resources it contains [6]. Anaerobic digestion’s low environmental impact and excellent energy recovery rate make it the most popular option for sludge disposal, and it can also help to reduce human dependence on fossil fuels. Traditional methods of sludge disposal, such as disposal in sanitary landfills, incineration, and incorporation into building materials, are not very effective, and the high energy consumption involved in these processes leads to the pollution of groundwater and the atmosphere [7]. Improving the efficiency of sludge resource utilisation and producing high-value-added products via the anaerobic fermentation of sludge has several economic advantages, including the simplicity of the required equipment, low operating costs, and the stability of its products. Anaerobic fermentation is the best and most practical method for converting biomass energy from an energy utilisation standpoint, as shown in Table 1. This article conducts a comparative analysis of various resource-based pathways utilised in the synthesis of medium-chain fatty acids (MCFAs), short-chain fatty acids (SCFAs), H_2_, and CH_4_. Additionally, it emphasises the present state of research and the manufacturing process of MCFAs.

## 2. Current State of Research on the Anaerobic Fermentation of Sludge

As a clean energy source, H_2_ promotes renewable energy use through the integration of energy storage and fuel efficiency, effectively helping to slow down environmental pressure. Compared with other chemical fuels, H_2_ has a high calorific value, high energy density, high power density, strong renewable characteristics, and it is convenient to transport. Dark fermentation, optical fermentation, and the use of microbial electrolytes (MECs) are the main methods of obtaining H_2_ from the anaerobic fermentation of municipal sludge. Among them, dark fermentation is considered the method with the greatest potential for biomass conversion via hydrogenation [12], and it has simple operating conditions. The advantages of stable hydrogen production and low energy consumption offered by this method give it vast development prospects. However, due to the constraints of current anaerobic sludge fermentation systems, some limitations exist in the production of biological hydrogen at an industrial scale, such as the low conversion rate of hydrogen production [13]; due to thermodynamic limitations, H_2_ is difficult to separate from CO_2_, resulting in a low yield of H_2_ [2]. Therefore, the production of H_2_ from municipal sludge is far from reaching industrial scales. Moreover, CH_4_ is also used as a renewable energy, as it has a high calorific value and can be used as a fuel or converted into electrical energy. At present, the industrial technology for producing CH_4_ is relatively mature, but CH_4_ has low efficiency and poor stability. During the production process, hydrogen sulphide and volatile silicon compounds are produced, which hinders the further development of the CH_4_ industry and greatly reduces the economic value and use potential of CH_4_ [14]. The SCFAs produced during the sludge digestion process can be added directly to nitrification systems as a carbon source, which not only reduces the investment cost of additional carbon sources, but also reduces sludge emissions, which is a very important application advantage. However, SCFAs are difficult to separate due to their poor hydrophobicity. MCFAs are highly hydrophobic and have low solubility, and the addition of acids to control them at a lower pH or near their pKa allows for MCFAs to exist in an undissociated state, floating on the surface of the solution in an oily form, thus spontaneously separating MCFAs from water [15]. 

**Figure 1:**
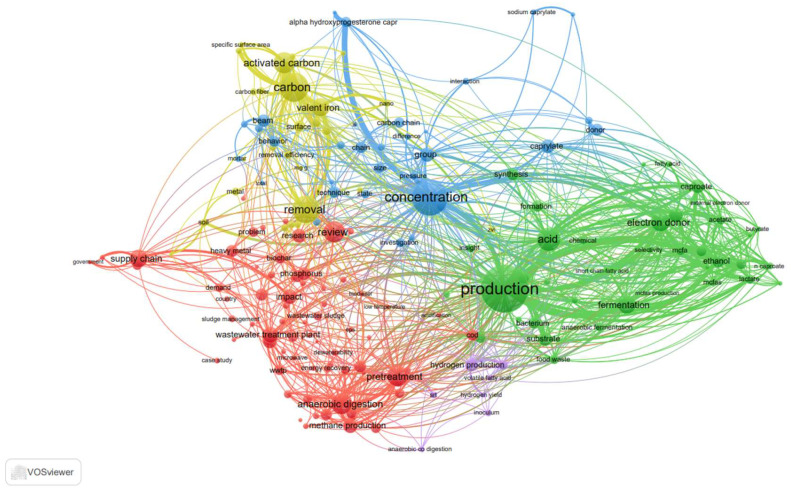
Overlay visualisation of utilisation of municipal sludge anaerobic fermentation resources (the size and colour of the nodes indicate correlations and different fields of immobilization, respectively).

Compared to H_2_, CH_4_, and SCFAs, MCFAs are of higher value and have a wider range of applications. For example, MCFAs can be used as medical and agricultural antibacterial agents [16]; in perfumes, food additives, lubricants, tobacco, spices, rubber, and dyes [17]; and as a fuel in aviation as a form of renewable diesel [18]. More importantly, the MCFA market is highly valuable; it is estimated to produce 25,000 tonnes annually, with a value of USD 1000 per tonne in a raw state and USD 2000–3000 per tonne when refined [19]. The market for MCFAs is growing globally and, as of 2023, it was valued at roughly USD 8 billion [20]. Today, chain elongation (CE) technology is economically viable, and CE methods have been shown to enable two important features in the industrial production of MCFAs: deterministic control of the reactor microbiota, thus increasing the efficiency of CE, and long-term operational stability [21]. In order to obtain pure MCFAs for potential use as additives in animal feed, a Dutch facility by the name of ChainCraft produces MCFAs from food waste. The facility is outfitted with downstream equipment for the separation and purification of MCFAs into liquid and powder forms [22]. Moreover, the acid whey produced by a Greek yoghurt plant in the state of New York yields about 8620 tonnes of MCFAs yearly, bringing in at least USD 32 million in income [23]. By integrating short-chain carbon resources into medium- and long-chain fatty acid resources, the carbon sources can be enriched and upgraded, which represents an important direction for the future development of sludge resources. Figure 1 depicts a variety of research co-occurrence networks for sludge resource utilisation (based on statistical results retrieved online by searching for anaerobic fermentation, pretreatment to produce MCFAs, electron donors (EDs), and CE enhancement). The development of sludge resources is introduced in the following sections. Research into sludge treatment, the main methods of producing MCFAs, and the comparison of different EDs strengthens the research on MCFA production.

## 3. Anaerobic Fermentation to Produce MCFAs

### 3.1. Research Related to Pretreatment

**Figure 2:**
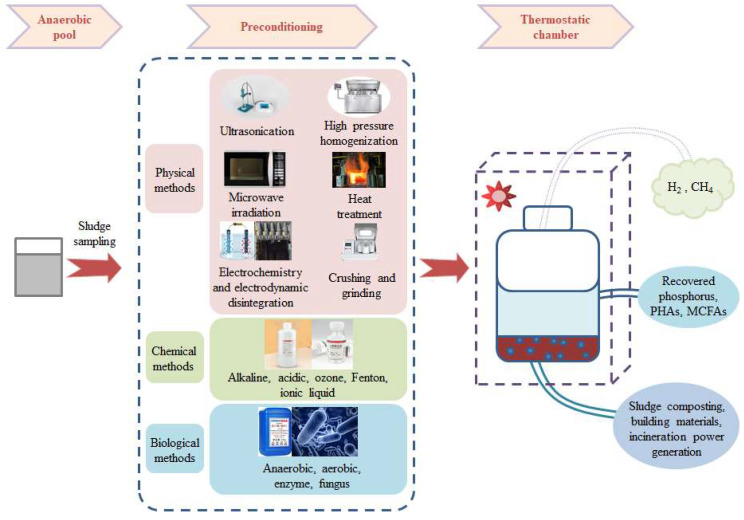
Pretreatment methods for enhancing anaerobic fermentation [27,28].

As a mature and effective sludge treatment technology, sludge anaerobic digestion uses anaerobic microorganisms to break down the complex and organic macromolecules present in sludge into small, stable molecules of H_2_, CH_4_, or SCFAs, and then recycles them. Alternatively, SCFAs can be used to further synthesise MCFAs, yielding three main advantages: sludge mass reduction, energy recovery, and environmental protection (Figure 2) [24].

In the process of the anaerobic digestion of sludge, hydrolysis is the main speed-limiting step. Due to the complex floc structure and hard cell walls of sludge, the degradation rate of organic matter is low. Complex sludge flocculants are treated to rupture the walls of microbial cells so that the organic matter of difficult-to-degrade particles is released into the liquid phase and converted by the process of biodegradation [25]. In addition, pretreatment will alter the microorganisms in the anaerobic fermentation system in a way that promotes SCFA production [26]. Whether acid pretreatment or the preparation of H_2_ and CH_4_ are applied, pretreatment is able to promote the hydrolysis of sludge; however, some pretreatment methods can inhibit the production of methane and increase the output of FAs. Therefore, this paper mainly discusses pretreatment methods that are conducive to the production of MCFAs and provides a reference for their production via the anaerobic fermentation of sludge.

The pretreatment methods for enhancing anaerobic fermentation are as follows: physical pretreatment, chemical pretreatment, biological pretreatment, and combined pretreatment. Among them, ultrasonic treatment is an effective pretreatment method. Its simple operation, non-harmful emissions, and improved sludge dewatering performance have made it one of the most extensively research methods [29,30]. In their experiments, Liu et al. (2018) found that when the ultrasound density was 2 W/mL and the ultrasound time was 15 min, the maximum SCFA output increased by 65.3% compared with no ultrasonic treatment [31]. 

Chemical pretreatment accelerates sludge hydrolysis by introducing acids, bases, and oxidants to break down organic molecules [32]. More research on acidic and alkaline treatments has been published in recent years. These treatments change the bacterial flora in sludge anaerobic fermentation systems, which changes the interactions between microorganisms, alters the flora’s function, produces metabolites, and ultimately alters the components of the products of this process. The great efficacy and ease of use of the alkaline treatment presented in [29] make it a solid choice. The advantages of alkaline treatments, such as their low energy usage, ease of operation, and inexpensive equipment requirements, have led to much research into these methods. Through solubilization and saponification, alkaline treatment kills the microbial cells and extracellular polymers in the sludge. It also improves the sludge hydrolysis rate by increasing the substrate’s interaction with anaerobic microbes [33]. A large number of studies have been conducted on alkaline treatments. At present, most of this research has focused on improving sludge anaerobic fermentation through the use of alkaline treatment in conjunction with other techniques. One study by Pang et al. (2022), for instance, indicated that both the sludge hydrolysis rate and SCFA synthesis were enhanced following pretreatment with an alkaline hydrolase mixture (pH = 10) that included alkaline protease, alkaline amylase, and alkaline cellulase [34]. For sludge containing wood fibres, acid treatment improves the anaerobic fermentation process because hydrolytic bacteria are better suited to acidic environments. However, this method is not as commonly used in sludge hydrolysis compared to alkaline treatment due to its potential destruction of downstream products, high cost, and the loss of fermentable sugars from complex substrate degradation [32,35]. In addition to acid–base processing, Xu et al. (2021) added 0.1 G/TSS KMnO_4_ for preprocessing, achieving a maximum SCFA output of 251.8 mg/g VSS. Through mechanism analysis, it was found SCFA generation provides more substances [36]. Xi et al. (2023) employed electrochemical preprocessing at 1.0 A using NaCl 1.0 g/L as the electrolytic solution and with a preprocessing time of 60 min and found that the treatment effect was significantly increased by 51.6% [37].

Biological pretreatment is a greener alternative to traditional pretreatment methods. It involves the use of a complex microbial substrate in which microorganisms break down the flocculent structure of organic compounds and sludge in various ways. Anaerobic pretreatment involves treating sludge at medium or high temperatures without the use of aerobic microorganisms, whereas aerobic pretreatment makes use of aeration [38]. The fast hydrolysis rate, energy savings, etc., are some of the advantages of enzyme treatment, where the organic macromolecules in the sludge are released from the substrate following the addition of hydrolytic enzymes. This process also offers promising future prospects [39]. Nevertheless, sludge hydrolysis is affected differently by various enzymes. Future studies should also focus on the selection of appropriate enzymes to improve the anaerobic fermentation process, as different enzymes have varying impacts on sludge hydrolysis. 

Pretreatment significantly improves sludge hydrolysis. Research into the optimisation and co-application of pretreatments is rapidly gaining momentum due to the following problems: the high energy requirements of physical pretreatments; the environmental hazards posed by chemical pretreatments such as acids, alkalis, and oxidants; the slow reactions and long cycle times of biological pretreatments; and the limited improvement in sludge hydrolysis achieved using a single pretreatment method. To address the first issue, we need to learn more about the chemical and physical properties of sludge, as well as the pretreatment conditions and process parameters used during fermentation, so that we can develop realistic and affordable pretreatment strategies. To improve anaerobic digestion and sludge disintegration, two or more pretreatments are usually used. For example, ultrasonic, alkaline, and other methods are combined, as shown in Table 2. At present, the literature focuses mainly on the effects of pretreatment on the effects of sludge hydration. Pretreatment methods lead to changes in the microbial community and thus to the development of anaerobic fermentation in favour of SCFA production. For example, Mirmohamadsadeghi et al. (2021) found that the selection of different pretreatment methods was found to alter microbial communities [40]. Thermal pretreatment of the inoculum could exterminate most of the initial methanogens and retain the CE-related bacteria [41]. In the study of SCFA production by dilute sulphuric acid pretreatment, Liang et al. (2024) found that the sulphuric acid concentration significantly affected the microbial community and led to the alteration of SCFAs [42]. In the future, the impact of the changes in the microbial community following pretreatment should be appropriately focused on, and pretreatment methods that are more suitable for strengthening the oxygen fermentation process of sludge should be sought out. 

### 3.2. The Typical Pathways for Generating MCFAs

SCFAs have two to five carbon atoms. They can be produced synthetically from fossil fuels or metabolically, with a maximum conversion rate of about 70%, from intermediates (proteins, polysaccharides, and lipids) produced during anaerobic digestion via fermentation and hydrolysis [48]. Unfortunately, the poor hydrophobicity and difficult separation and purification of SCFAs restrict their applicability. MCFAs with six to twelve carbon atoms have a higher energy density but, because of their longer carbon chains, they are also more hydrophobic. As a result, a specific concentration of MCFAs floats on the reaction solution’s surface as an oily liquid, which indicates that MCFAs separate from water on their own [49]. In addition, their solubility decreases dramatically as the carbon chain lengthens (from 10.82 g/L at C6 to 0 g/L at C10), making separation and extraction processes easier [22]. An alternative method for the biotransformation of SCFAs is offered by the CE process, a major metabolic pathway for the microbial synthesis of MCFAs. This process uses the fatty acid biosynthesis (FAB) pathway or reverse β-oxidation (RBO) pathway with SCFAs as the electron acceptor and ethanol, lactate, syngas, etc., as the ED.

**Figure 3:**
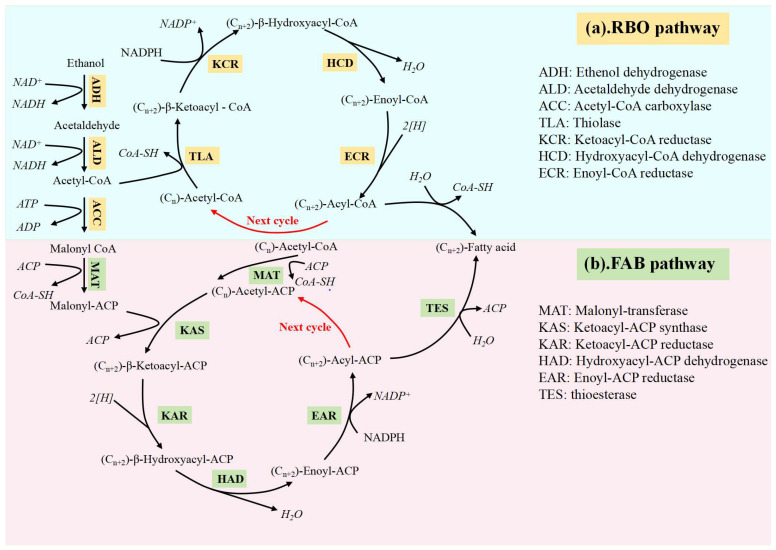
The typical pathways for MCFA production [22].

Recently, considerable effort has been devoted to the synthesis of MCFAs with the aim of facilitating their widespread implementation. In the process of CE, functional microorganisms mainly metabolise ethanol or lactate, and the RBO pathway is preferentially lower than the ED containing ethanol or lactate, and the preferred substrate of the FAB pathway is carbohydrates such as lignocellulosic biomass. Therefore, although the FAB pathway is widespread, the RBO pathway is the main acid production pathway in the carbon chain extension of sludge substrate, and therefore the RBO pathway is more favourable for acid production [49,50]. This fact enhances the potential of CE technology and provides an opportunity to further refine the production of MCFAs [22,51]. Thus, in conjunction with the comparison of different EDs, our main focus was to investigate the RBO pathway (Figure 3a) and the FAB pathway (Figure 3b) of MCFA production using ethanol as an ED in open culture systems. In both pathways, acetaldehyde is converted to acetyl-CoA via acetaldehyde dehydrogenase; approximately one-sixth of acetyl-CoA is acetaldehyde.

For example, in the reverse beta-oxidation cycle, acetate can be coupled with butyrate, butyrate with caproate, valerate with heptanoate, etc., in order to produce a new coenzyme that is a derivative, with two additional carbon atoms in the chain [52]. The main factors influencing the even chain are the differences in substrate and inoculum. When propionate or propiogenic bacteria dominate the substrate, the concentration of heptanoate is higher. Kim et al. (2019) found that when propionate was added, valerate and heptanoate were produced at relatively high concentrations [53]. With eight carbon atoms, caprylate has the most carbon atoms that can currently be obtained, although it can be expanded to a maximum of 12 carbons. This is because the product’s toxicity increases as the number of carbons increases [21]. The conversion of acetate to butyrate involves a sulfurylase called acetyl-CoA, which couples two acetyl-CoA molecules to form acetoacetyl-CoA. The next step in the production of butyryl-CoA involves a series of enzymatic processes. The reaction between butyryl-CoA and acetate produces butyrate and fresh acetyl-CoA. Ethanol oxidation produces more acetyl-CoA, which reacts and extends the chain again to form acetate, after which the freshly released acetyl-CoA is bound to it. Additionally, the cycle of butyrate’s elongation to caproate is initiated when acetyl-CoA binds to butyryl-CoA [15].

Each cycle adds two carbon atoms, but the FAB pathway is more active and consumes more ATP. Both the FAB and RBO pathways depend on acetyl-CoA to initiate the cycling process [54]. The FAB cycle begins with the production of acetyl-CoA by ED, acetyl-CoA in the presence of acetyl-CoA carboxylase (ACC), and the consumption of ATP to produce malonyl-Coenzyme A (malonyl-CoA). Then, in the presence of ketoacyl synthetase (KAS), there is an initial condensation reaction between acetyl-CoA and malonyl-ACP to produce acetyl-ACP. Acetyl-ACP is then reduced to butyryl-ACP [55] and butyrate [56] through the action of acyl-ACP thioesterase (TES). In this particular cycle, butyryl-ACP is converted to hexanoyl-ACP, which has two extra carbon atoms in its chain and is then hydrolysed by TES to become caproate [57]. To enter this cycle, butyryl-ACP is first condensed with additional malonyl-ACP molecules by ketoacyl synthase (KAS). In terms of energy synthesis, one molecule of ATP is consumed for each molecule of malonyl synthesised, and the FAB pathway requires more ATP than the RBO pathway, resulting in lower MCFA production efficiency [58]. 

### 3.3. Comparison of EDs

The driving force for CE reaction systems comes from EDs, which are reducing chemicals that are rich in energy. The CE pathway, final product type and yield, and the specific ED used are all dependent on the specific oxidation processes used. Among the many EDs discovered by Chen et al. (2016) for CE in batch and continuous operations is methanol, joined by ethanol and lactate [59]. Using CO as the only substrate, He et al. (2018) discovered that MCFAs could be synthesised without increasing the number of EDs [60]. In their study, Veras et al. (2020) used glycerol, a by-product of biodiesel production, to produce valerate [61]. When the ratio of propanol and acetate was close to 1:1, the concentration and selectivity of heptanoate were the highest. On the other hand, ethanol and lactate are the best EDs. Due to their high reducing equivalents and ease of conversion to acetyl-CoA, ethanol and lactate were found to be the best EDs for the synthesis of MCFAs [21]. Therefore, the electron-donating chemicals ethanol and lactate and the functional microbes that play a role in this process are the primary targets of research in this area.

One of the most studied EDs in CE systems is ethanol. Yeast fermentation, syngas, and distillation are all viable options for producing ethanol, which is a popular practice in the biofuel sector [62,63]. However, these methods of ethanol production are quite expensive. Therefore, in real CE systems, there is an opportunity to reduce resource consumption during ethanol recovery, make better use of organic waste resources, and achieve greater economic benefits by directly using ethanol-containing organic waste, such as brewery sludge, to produce MCFAs.

For ethanol, the predominant ED mechanism is the reverse RBO pathway. Through experimentation, Reddy et al. (2018) discovered that, during CE, *Clostridium kluyveri* (*C. kluyveri*) converts acetate and ethanol into compounds such as butyric and caproate [64]. *Clostridium* IV and *Clostridium sensu stricto* were the predominant strains for CE in Wu’s study [65]. In pure bacterial experiments using ethanol as an ED, *C. kluyveri* was the most commonly used strain and the first strain found to have the best effect on CE. The reaction conditions when using ethanol as an ED are also crucial to the CE process. The ideal initial pH range for *C. kluyveri* using ethanol and acetate as substrates is between 6.6 and 7.5 [66]. When n-butyrate was synthesised from acetate, with ethanol serving as the ED at pH = 7 and 25 °C, no thermodynamic bottleneck was observed. The RBO pathway did not occur in the bioreactor at 55 °C, but it did occur at 30 °C, suggesting that a thermodynamic bottleneck or product toxicity may be limiting CE at higher temperatures [67]. Most studies found that the ideal conditions for producing MCFAs were pH = 7 and 30 °C, which is quite close to the ideal pH (pH = 6.8) and temperature (34 °C) for *C. kluyveri* [66]. As the ratio of undissociated/dissociated MCFAs was significantly higher at pH 7, and the methanogenic bacterial activity was suppressed the most at pH 5.5, in-line extraction was required to produce significant isocaproic acid concentration. Additionally, in-line extraction can prevent product and substrate toxicity [49]. Numerous research studies have indicated that one of the most promising approaches for the future commercialisation of MCFAs is a production strategy that combines an in-line extraction unit with a lower pH (pH = 5.5) [21]. Apart from the impact of pH, CO_2_ has an impact on the microorganisms involved in the CE process. In order to avoid toxicity to microorganisms and to obtain higher concentrations of MCFAs, it is usually necessary to use an additional carbon source to keep the reaction at a lower ethanol concentration. Additionally, the mixed culture must be maintained at a high partial hydrogen pressure to prevent the degradation of carboxylates [68,69,70].

There are further studies on the role of ethanol as an ED in the CE process, but other literature suggests that lactate and carbohydrates will be the preferred EDs in future studies and industrial production, with the use of ethanol being minimized [71]. The fact that lactate can be converted to caproate was first discovered by Elsden et al. (1956) [72]. It was once thought that the dominating genus for converting lactate to caproate was *Megasphaera elsdenii* [73]. However, more in-depth research led to the discovery of *Clostridium* VI [70], *Acinetobacter* spp. [74], *Ruminococcaceae* CPB6, and other species that are involved in the CE system [75]. Researchers have used cheese whey and sewage sludge co-fermentation in a continuous reactor to produce caproate and other VFAs [76], and they found that the microbial community was dominated by *Olsenella* sp. and *Rumodicacceae* sp. [77]. However, lactate can also be used as the sole carbon and energy source for the production of caproate via a unique microbiota obtained from a white wine cellar [70]. Recently, Liu et al. (2020) discovered three new strains of *Clostridium* spp. (BL3, BL4, and BL6) that are also capable of converting lactate into hexanoic and isobutyrates [76]. The ability to add higher concentrations of lactate without toxic inhibition of microorganisms and the absence of exogenous CO_2_ supplementation are the advantages of lactate over ethanol as an ED; however, the carbon source is diverted in the form of CO_2_, and the acrylate pathway also contributes to the diversion of the carbon source, resulting in a lower MCFA concentration. The mechanism of the lactate CE reaction consists of three primary components: lactate oxidation, the acrylate pathway, and the RBO cycle. The lactate oxidation and RBO cycle compete with each other, resulting in a lower concentration of MCFAs [73]. Therefore, changing the reaction conditions should increase the concentration of MCFAs. A higher pH is more advantageous for the synthesis of propionate and, despite the higher rate of lactate consumption, the synthesis is faster and a lower pH (pH < 6) is more favourable for the formation of caproate from lactate [78]. The CE of butyrate with lactate between pH 4.5 and 5.0 has been shown to give the highest concentration of heptanoate. Additionally, some researchers have found that supplementing butyrate or acetate during the CE process with lactate as an ED can help to reduce the amount of lactate and the lag time in the production of MCFAs [75]. Nzeteu et al. (2022) used acidified mixed anaerobic sludge as an inoculum to efficiently produce 91.7 ± 0.5 mM caproate at pH = 3 and a substrate concentration of 300 mM (lactate/butyrate = 1:1), with a selection rate of over 90% [79]. Lactate can be used as the sole ED or as a carbon chain extender with ethanol and fructose acting as EDs to enhance the production of MCFAs. Kang et al. (2022) found that the concentration of caproate produced during fermentation was 8.9 g/L when lactate was the only ED utilised, and this was 20.9 g/L when fructose and lactate were simultaneously employed as the EDs [80]. Wu et al. (2018) found that ethanol and lactate as co-EDs could resolve the respective shortcomings of a single ED: ethanol could elongate the lactate-derived propionate to decrease lactate–carbon diversion, and lactate oxidation could supplement the CO_2_ required for the ethanol-driven CE [81]. Future research could investigate the combination of lactate and other EDs, such as ethanol, fructose, glycerol, etc., to increase concentration of MCFAs. The use of lactate as an ED for CE has both economic and environmental benefits.

### 3.4. The Technique for Enhancing Product Synthesis

The main problems preventing anaerobic fermentation from producing sufficient amounts of MCFAs are low acid output and an unstable acid production process. Avoiding the product inhibition and toxicity associated with dissociated carboxylic acids is crucial because, during anaerobic fermentation, undissociated MCFAs pass through the cell membrane and dissociate in the cytoplasm, releasing protons and limiting growth [65]. The most commonly used ED, ethanol, also has an inhibitory effect on the CE reaction mechanism in addition to undissociated acids. Lonkar et al. (2016) discovered in their experiments that higher ethanol concentrations (40 g/L) completely suppress microorganisms, thus inhibiting the carbon chain extension reaction system [82]. In addition, Wu et al. (2019) found that timely extraction of MCFAs is the best way to prevent product suppression [15]. Kucek et al. (2016) tried to control the toxicity of the product and found that the concentration of the caprylate was 0.33 g/L [18]. Hernandez et al. (2021) extracted and separated MCFAs from a synthetic fermentation solution via a membrane electromagnetisation method, and the total recovery of carboxylic acid salt was 60 ± 3% [83]. However, using in-line extraction devices is quite expensive. Thus, it is more pertinent to identify a low-cost way to boost the microbial metabolism in the CE reaction system. In recent years, carbon materials have been widely studied due to their convenient preparation technology, low cost, and significant improvement of anaerobic fermentation efficiency. Whether they take the form of biological carbon, activated carbon, or carbon nanotubes, carbon materials have high stability and large ratios. Their large surface area, good adsorption ability, and high conductivity have made carbon materials the best choice for environmental remediation. In recent years, carbon materials have also started to be used for anaerobic CH_4_ fermentation. It has been found that carbon materials can promote species transfer between bacteria and methane bacteria, direct electronic transfer, increase buffering capacity, alleviate the suppression of SCFAs and ammonia nitrogen, and enhance the diversity of paleo-biological communities, thus improving the efficiency of CH_4_ production [84,85]. This also provides further direction for extending and strengthening the carbon chains of sludge. Liu et al. (2020) discovered that the CE reaction system was successfully reinforced by the inclusion of biochar particles smaller than 5 mm, which also decreased the reaction time and increased the selectivity of caproate up to as high as 93.56% [86]. Wu et al. (2024) studied the effect of different particle sizes of zero-valent iron (ZVI) on sludge production of fatty acids, and 75 μm was found to be more beneficial to the production of MCFAs. Through the analysis of functional enzymes, it was found that ZVI can enhance the β-antioxidant pathway [87]. In addition to carbon materials, ZVI and nano zero-valent iron (NZVI) are used to strengthen MCFA additives for anaerobic fermentation due to their advantages of being low cost, easily available, and environmentally friendly. ZVI was used by Wang et al. (2020) to increase the production of MCFAs. At doses of 1–20 g/L, ZVI effectively increased the concentration and selectivity of MCFAs; when 20 g/L was added, the concentration of MCFAs reached 15.4 g COD/L and the selectivity reached 71.7%. ZVI also has the ability to transfer electrons, and caproate has a selectivity of up to 93.56%, which successfully improved the CE reaction system [88]. ZVI can also enhance sludge hydrolysis and acidification by transferring electrons, increasing the amount of substrate available for MCFA synthesis. By raising the system’s pH and conductivity and lowering its ORP, the addition of ZVI enhanced the efficiency of electron transport and improved the conditions for biological anaerobic activity [89]. The addition of ZVI increased the pH and conductivity of the system and improved the efficiency of electron transport. Fu et al. (2021) discovered that, in addition to ZVI, NZVI could also significantly increase the concentration of caproate. When 5 g/L NZVI was added, the caproate concentration increased to 27.2 mmol/L, which was over 100% higher than the control [90]. By exerting its selective reducing action, NZVI altered the bacterial population by promoting the synthesis of H_2_, increasing the number of EDs available to support the flow of electrons to the longer carboxylic acid chain products [91].

Why is NZVI effective at increasing MCFA production? This question has received explanations from numerous researchers. NZVI is able to work via the following displacement reaction: $Fe+2H^{+}\to {Fe}^{2+}+H_{2}$. In order for *C. kluyveri* to participate in the CE process, the pH must not go below 5.4. NZVI can also supply extra electrons by promoting the oxidation of ethanol, which promotes the synthesis of caproate [90]. NZVI was found to act as a pH buffer to improve the CE process, helping to contribute to the functional stabilisation of the microbial community and reduce the problem of product inhibition. NZVI also corrodes to produce H_2_, which can be used as an ED [92]. Additionally, NZVI increases the activity of ferredoxin oxidoreductase, which can be added to microbes to enhance ferredoxin synthesis and thereby promote product CE [90,93]. NZVI has been widely accepted as a reinforcing material for CE systems because it is more reactive and dispersible than ZVI, has a larger specific surface area, and releases more H_2_ more quickly. In future research, as different mechanisms of carbon materials may be different, the most appropriate carbon materials should be selected as reinforcement materials for different substrates. The changes in acidic bacteria and their effects on the type of enzyme produced can improve the mechanism of action in the anaerobic fermentation of MCFAs. In addition, in order to establish a relationship between the performance of reinforcement materials and microbial changes, different materials should be explored for different inoculations, especially concerning their mechanisms of action during anaerobic fermentation, combinations with microorganisms, changes in functional enzymes, and improvement in the amount of materials added. Research in this direction is needed to obtain optimized methods for fatty acid production.

### 3.5. MCFA Production Constraints and Solutions

Currently, the main challenge in the process of sludge fermentation production of MCFAs is the low concentration and yield of MCFAs. The main reasons are incomplete substrate utilisation, competitive reactions, product inhibition, and difficulty in separation and extraction [11]. For incomplete substrate utilisation, pretreatment should destroy the structure of the sludge so that acid-producing microorganisms can better utilise the organic matter in the sludge, and pretreatment can also inhibit the growth of methanogens. There is also the use of multi-stage reactors to improve the utilisation efficiency of sludge. Wu et al. (2021) achieved a maximum MCFA yield of 67.39% using a two-stage reactor [65]. Competition reactions mainly involve substrate consumption from methanogens and sulfate-reducing bacteria and excessive ethanol oxidation to acetate. The current main methods are to add methanogen inhibitors such as 2-bromoethanesulfonic acid (2-BES) and CHCl_3_ [94]. To avoid excessive oxidation of ethanol, the most environmentally friendly method is to increase the partial pressure of hydrogen (at least 0.03 atm above). For product inhibition, in addition to appropriately increasing pH to reduce the concentration of undissociated MCFAs and mitigate the toxic effect of undissociated acid, the addition of biochar to adsorb microorganisms to form granular sludge can help microorganisms to resist toxic substances and improve the efficiency of electron transfer [95]. In addition to biochar, iron materials such as ZVI and Fe_2_O_3_ can be added to promote electron transfer between species and increase the concentration of MCFAs, as shown in Table 3. Nonetheless, the most fundamental method is to extract the product in time. At present, the main uses of liquid–liquid extraction, membrane separation, electrodialysis, adsorption, straight distillation, and chemical precipitation, have been to recover undissociated carboxylic acids from the fermentation systems of waste streams [22]. Grootscholten et al. (2013) found that the yield of caproate was 57.4 g·L^−1^·d^−1^ using a continuous feed reactor, and Xu et al. (2021) developed an electrodialysis/phase separation cell with an oil flux of 1.66 kg·d^−1^·m^−2^ for MCFAs [96,97]. However, in the extraction of low-concentration MCFAs, the power consumption of the electrodialysis/phase separation cell will be greatly increased, and therefor more research is needed in the future to overcome the difficult problem of product separation and extraction.

## 4. Conclusions and Future Prospects

Similarly to other anaerobic fermentation technologies, the production of MCFAs from municipal sludge via CE has certain constraints. Further studies are required to support the advancement of such systems. Further expansion of MCFA production and additional products of CE processes are constrained by the harmful effects of the substrates used and the by-product inhibition of the microbiota used in CE systems. In order to increase the yield of MCFAs, researchers must take appropriate steps to mitigate the inhibitory effects of toxicity. In order to increase the overall efficiency of the CE reaction system, it will be crucial to investigate how to effectively overcome the problem of product and substrate inhibition, such as by adding functional enhancement materials or refining the form of the reactor. Furthermore, caproate makes up the majority of the produced MCFAs in the current study, but caprylate and heptanoate are more hydrophobic, easier to separate, and more cost-effective. In the future, obtaining products with longer carbon chains will also become a significant area of research.

The carbon neutrality concept has brought increased attention to the use of sludge resources. In addition to reducing the reliance on fossil fuels, the production of H_2_, CH_4_, and MCFAs from municipal sludge can help to mitigate environmental damage and, to some extent, lower greenhouse gas emissions. However, H_2_ and CH_4_ have a comparatively limited application channel and their economic values are low. On the contrary, the MCFAs produced via the CE pathway from sludge not only have a variety of commercial uses, but they can also be used as fuel precursors for the production of liquid fuels, realising the high-value resource utilisation of bulk biomass waste. This process can also support the circular bioeconomy, a concept that aims to address the three main issues of greenhouse gas control, waste recycling, and fossil fuel substitution. 

## Figures and Tables

**Table 1:** Comparison of thermochemical technologies for sewage sludge disposal and resource exploitation [8,9,10,11].

Resource Technology	Process Temperature (°C)	Products	Advantages	Disadvantages
Incineration	˃850	Electric energy	Reduces 90% of sludge volume with the simultaneous destruction of pathogens	Produces harmful gases, high investment cost
Sludge composting	-	Organic fertilizer	Provides nutrients for plant growth	Low efficiency, long cycle required
Gasification	Up to 1100	Syngas (H_2_, CO, CH_4_, C_x_H_y_)	Produces combustible gases for energy recovery	Environmental pollution risk
Pyrolysis	400~800	Syngas (H_2_, CO, CO_2_, CH_4_, C_1_–C_4_)	Reduces 50% of volume and all byproducts have energy and circular economy value	Environmental pollution risk
Anaerobic digestion	-	MCFAs	Widely used, high economic value	Separation and purification process is not perfect
Anaerobic digestion	-	H_2_	As a clean energy, alleviates the greenhouse effect	Low efficiency
Anaerobic digestion	-	CH_4_	Eases dependence on fossil fuels	Single application, compared with MCFAs economic value is low

**Table 2:** A survey of combined sludge pretreatment methods.

Pretreatment Method	Pretreatment Conditions	Pretreatment Results	References
EDTA-2Na hydrolysis (EH) and protease hydrolysis (PH)	PH: 25 mg/L dry sludge EH: 0.2 g/L dry sludge Stirring speed: 120 rpm Temperature: 25 °C	SCFA concentration of 12,704.44 mg COD/L, about 9.3 times the control WAS without pretreatment	[43]
Alkaline protease (AP) and alkaline treatment	AP: 5% g/g VSS pH: 10 Pretreatment time: 2 h Temperature: 35 °C	SCFA concentration of 607 mg COD/g VSS was produced over an anaerobic fermentation of 3 days, which was 5.4 times higher than the control	[44]
Free ammonia (FA) and aided ultrasound pretreatment	Ultrasound (2 w/mL): 15 min FA (60 mg/L): 2 d	SCFA concentration of 316.7 mg COD/g VSS, which was 1.7 times higher than that of pretreatment using ultrasound (FA) alone	[45]
Sodium dodecyl sulphate (SDS) and mixed enzymes	SDS: 0.20 g/g dry sludge Enzymes: 0.06 g/g dry sludge (protease: a-amylase = 3:1) Temperature: 50 °C	SCFA concentration increased by 1.82 (6th day), 2.04 (5th day), and 2.32 (7th day) times	[46]
Free nitrous acid and alkaline treatment	pH: 10 Free nitrous acid: 1.54 mg/L Pretreatment time: 2 d Temperature: 20 °C	SCFA concentration of 370.1 mg COD/g VSS, which was 4.7 times higher than the control	[47]

**Table 3:** Comparison of MCFA production with different materials.

ED Level (Ethanol)	Conditions	Strategy	Maximal MCFA Production	References
140 mmol/L	Initial pH = 5.5 Temperature = 37 °C	20 g/L ZVI	15.4 g COD/L	[88]
170 mmol/L	Initial pH = 6.9 Temperature = 35 °C	20 g/L Fe_2_O_3_	9162 mg COD/L	[98]
360 mmol/L	Initial pH = 7 Temperature = 36 °C	10 g/L ZVI (75 µm)	4782.88 mg COD/L	[99]
50 mmol/L	Experiment used upflow blanket filter reactors Temperature = 35 °C	20 g/L biochar	46.69 g COD/L *	[100]
140 mmol/L	Initial pH = 7 Temperature = 35 ± 2 °C	5 g/L Fe_3_O_4_ (20 nm)	9614.26 mg COD/L	[101]
170 mmol/L	Temperature = 35 °C	20 g/L Fe_3_O_4_	7953.6 mg COD/L	[102]

* Denotes the concentration of caproate.
